# Addressing Disparities in Alzheimer's Disease and African-American Participation in Research: An Asset-Based Community Development Approach

**DOI:** 10.3389/fnagi.2019.00125

**Published:** 2019-05-31

**Authors:** Gina Green-Harris, Sheryl L. Coley, Rebecca L. Koscik, Nia C. Norris, Stephanie L. Houston, Mark A. Sager, Sterling C. Johnson, Dorothy Farrar Edwards

**Affiliations:** ^1^Wisconsin Alzheimer's Institute Regional Milwaukee Office, University of Wisconsin School of Medicine and Public Health, Milwaukee, WI, United States; ^2^Center for Community Engagement and Health Partnerships, University of Wisconsin School of Medicine and Public Health, Madison, WI, United States; ^3^Wisconsin Alzheimer's Institute, University of Wisconsin School of Medicine and Public Health, Madison, WI, United States; ^4^Geriatric Research Education and Clinical Center, Wm. S. Middleton Veterans Hospital, Madison, WI, United States; ^5^Wisconsin Alzheimer's Disease Research Center, University of Wisconsin School of Medicine and Public Health, Madison, WI, United States; ^6^Department of Kinesiology, School of Education, University of Wisconsin, Madison, WI, United States; ^7^Department of Medicine, University of Wisconsin School of Medicine and Public Health, Madison, WI, United States

**Keywords:** African Americans, Alzheimer's disease, health disparities, recruitment, community engagement

## Introduction

Alzheimer's Disease and related dementias (ADRD) remain a worldwide burden to address for persons with ADRD, their family members, and others at risk. ADRD should be recognized as a public health priority (Barnes, 2019), particularly among African Americans for several reasons. Currently, African Americans have 2–4 times the risk of developing ADRD than non-Hispanic Whites (Barnes and Bennett, 2014; McDonough, 2017). Given the higher presence of co-morbid conditions and associated health disparities when comparing African Americans to other racial and ethnic groups (Dillworth-Anderson and Boswell, 2007; Hill et al., 2015), African Americans can have worse physical health and quality-of-life. These factors can also increase costs of care for persons with ADRD and their family members. African Americans could be further burdened by significantly higher costs of care than Whites (Gilligan et al., 2012) and lack of adequate insurance.

Despite these disparities, African Americans are still underrepresented in ADRD research. Cultural and institutional barriers include primary reliance on passive recruitment strategies (e.g., postings, flyers, database recruitment) not tailored to communities of color, implicit bias, and mistrust of medical communities, academic institutions, and researchers (Ballard et al., 2010; Scharff et al., 2010; Williams et al., 2010; Shin and Doraiswamy, 2016). Investigator inclusion and participation criteria deemed more feasible for White participants (ex. Medical eligibility criteria, job restrictions, time, and financial requirements to go to study sites) also undermine or limit the participation of African Americans and other participants of color (Wendler et al., 2006; Konkel, 2015).

Using asset-based community development (ABCD) approaches (Kretzmann and McKnight, 1993) can lead to improved access and delivery of care and ADRD knowledge among African Americans, increased social support for African-American caregivers and elders living with or at risk for ADRD, and increased African-American representation in neuroscience research. In this article, we describe the development and implementation of an ABCD approach for increasing African-American representation in the Wisconsin Registry for Alzheimer's Project (WRAP).

## WRAP and Initial Recruitment

WRAP started in November 2001 with participants initially recruited from adult children of parents with ADRD receiving care from Wisconsin Alzheimer's Institute (WAI) memory diagnostic clinics (Sager et al., 2005). WRAP remains one of the largest and longest-running longitudinal observational cohort studies for adult children of persons with Alzheimer's Disease (*N* > 1,100 with parental history of AD and >400 persons without known parental history) for identifying early declines in cognition and examining midlife factors and biomarkers related to the disease (Johnson et al., 2018). Initial recruitment strategies included educational presentations, clinic referrals, newspaper advertisements, and emails sent to University of Wisconsin-Madison (UW) alumni. Using these recruitment methods reached few African Americans, particularly alumni recruitment given the historically low rates of alumni of color at predominantly White universities including UW. Only six African Americans (0.8% of 711) enrolled in WRAP by 2005.

To address this recruitment disparity, strategies of recruiting from communities that are more diverse and hiring a more diverse study team were implemented. Specifically, Milwaukee became a recruitment site in 2006; African Americans and other residents of color constituted over 50% of Milwaukee's population (United States Census Bureau, 2006). A Milwaukee-based office was established and African-American program and outreach managers were hired to implement recruitment strategies in Milwaukee that were similar to those used in Madison (e.g., clinic-based recruitment and advertisements). African-American recruitment at the end of 2007 remained low (<2% of WRAP) despite implementing these strategies—changing recruitment strategies was identified as an important next step.

## WAI-Milwaukee Asset-Based Community Development Model

In 2008, new WAI-Milwaukee leadership who had lifelong connections to Milwaukee developed and implemented an ABCD approach to African-American recruitment. This approach patterns Kretzmann and McKnight (1993) ABCD community-engagement principles through connecting residents with culturally-tailored programs and services with community-identified needs. ABCD maintains that initiatives from exterior sources such as university-based research projects will be most effective when community assets are leveraged at full capacity. Sustainable community development stems from (1) assessing resources, skills, and experience available in community members, (2) organizing communities around issues that move members into action, and (3) community members determining priorities and taking appropriate action. This community development leads to increased services provided to communities and increased representation of African Americans in research. Table 1 (Panel A) illustrates the core aspects of WAI-Milwaukee's ABCD approach.

**Table 1:** WAI-Milwaukee Asset-Based Community Development (ABCD) Approach, examples of community activities and current outcomes.

**Panel A: Core Aspects of ABCD Approach**	**Panel B: Examples of Activities**	**Panel C: Outcomes**
Community assessment • Identify the community and their assets • Identify key community stakeholders • Address the community needs and wants	Outreach & Education • Breaking the Silence Annual Breakfast and Community Workshops • The “Amazing Grace” Chorus Family Support Program • Annual Faith-Based ADRD Initiatives	Stigma Reduction Increased community awareness of ADRD
Community engagement (WAI-Milwaukee becoming part of community “social fabric”) • Invest time in the community • Provide resources identified by the community • Address barriers through service	Coordination of Medical & Social Services Milwaukee Health Services Center & Clinic Network Development Culturally inclusive professional training	Increased access of community members to comprehensive care and support services
Community involvement • Recognize community members as experts • Validate community members' perspectives • Build relationships between community members, researchers, and health professionals • Community members provide counsel to WAI-Milwaukee and researchers	Community Advisory Board development and sustenance	Academic-community partnership in initiatives Increased community awareness of importance of research Increased African-American participation in research

Several strategies were initiated through this approach (Table 1, Panel B), with the identification of community assets and key stakeholders as the first step. Through grassroot networking efforts, the new leadership team identified key stakeholders among African-American residents affected by ADRD. This networking led to the identification of 10–15 African-American residents and caregivers with diverse roles in their respective communities. Stakeholders included an African-American physician, public health officials, and local media professionals who cared for family members with ADRD. The WAI-Milwaukee leadership team and stakeholders developed a community advisory board (CAB) in 2008 that counseled the WAI-Milwaukee team on culturally-tailored outreach and engagement, recruitment strategies, and potential resources that could be utilized for community-based efforts. Developing CABs is an effective strategy for overcoming barriers related to power differentials between researchers and communities in which the research is being conducted (Dancy et al., 2004).

Next, the Milwaukee Health Services Memory Diagnostic Clinic was developed with local infrastructure funding through the university's Wisconsin Partnership Program. Led by an African-American physician, it became the first memory diagnostic clinic in the nation located in a federally-qualified medical center. Memory clinic patients received complementary health education, dementia classes, and lifestyle intervention courses. Working with clinics within the WAI clinic network gave WAI-Milwaukee credibility in African-American communities for being a trusted resource of clear and relevant information.

This credibility provided a platform for WAI-Milwaukee to become part of the community social fabric (Jeste et al., 2016) to deliver patient-centered training and community resources to engage African-American elders. For example, CAB meetings revealed residents' desire for advocacy training for themselves and their family members to use during provider interactions. Provider education was also identified as a critical need given community members' reports of insufficient education received from providers about health conditions. Based on community feedback, primary care and allied health workers received information from WAI-Milwaukee about articulating health information back to patients with inclusion of family members and considerations for enhancing African-American patients' trust in the medical system.

For developing culturally-tailored programming, the WAI-Milwaukee team identified and applied community-based culturally-tailored aspects into educational initiatives originally created for mainstream audiences. Incorporating communities' value systems and historical perspectives means identifying the norms, continuously asking questions, and becoming educated by community members in a cyclical process. As a leadership team member, an African-American family care coordinator talked with families in their homes to identify barriers and connect families to services and programs while incorporating cultural respect (ex. Addressing titles, meeting them during times and events convenient for them). Through preliminary education sessions, community members expressed their readiness to talk about brain health-oriented solutions before discussing ADRD. Milwaukee CAB members facilitated these education sessions that enhanced the co-learning process between African-American residents and WAI-Milwaukee. With the support of reputable African-American organizations, these education sessions led to larger health promotion efforts in various residential areas of the city, including lower-income high-rise apartments. To address social support desires expressed by caregivers, programming for African-American caregivers and family members with dementia included the development of the Amazing Grace Chorus Family Support Program. The leadership team developed this program with local music teachers through adapting current mainstream music therapy interventions (Mittelman and Papayannopoulou, 2018) designed to improve quality of life and social support for the caregivers and family members with ADRD (Refer to Table 1, Panel B for more programming examples).

## WRAP Recruitment Lessons for Success

This reciprocal community-university partnership yielded favorable outcomes to date (Table 1, Panel C). Through this partnership, WAI-Milwaukee gathered valuable information on community priorities from African-American members for informing community-engagement activities, providing beneficial services for community members, and reducing stigma about ADRD. Subsequently, WAI-Milwaukee built trust among community members and providers and commitment to research. From 2008 to 2012, African-American participation in WRAP increased more than 400% from 0.8 to ~8.0% of the study sample (131 of 1,573). Recruitment of new African-American participants plateaued for several years when budget constraints allowed primarily for return visits of existing participants; recent funding allows resumed recruitment of more African Americans.

Several lessons are worth consideration for increasing African-American research participation. Although similar strategies have been used for aging health studies [ex. (Gluck et al., 2018)], our approach emphasizes meeting the community members' needs first through service before discussing participation in research. Through an iterative process with community members, the leadership team addressed other issues related to ADRD before explicitly addressing ADRD. Recruiting African-Americans to participate in research was the last step; notably, families agreed to participate in WRAP as reciprocity for the services received from WAI-Milwaukee. This approach continues as an iterative process to meet the needs of the communities and to retain members' interest and participation in research.

Investment in relationship building remains essential for recruitment (Barnes, 2019), and multiple stakeholders must have shared vision and mutual trust. CAB community members shared recruitment responsibilities with the WAI-Milwaukee team at community centers and events, and the WAI-Milwaukee team shared recruitment responsibilities with WAI clinic network physicians in the clinics. African-American recruitment was primarily word-of-mouth with the shared message that WRAP participation helps African Americans receive better clinical diagnoses, therefore African Americans would benefit from research participation. WAI-Milwaukee credits the communities for building the model for WRAP recruitment.

Next, alternate funding streams apart from government funding are feasible resources for community engagement infrastructure building and related activities that enhance research efforts. WAI-Milwaukee credits philanthropic efforts (Bader Philanthropies, Wisconsin Partnership Program) which allowed room for capacity-building, community engagement, and developing relationships outside of rigid timeframes of traditional research funding. Building the strategic framework and developing capacity is critical before focusing on obtaining research funding through traditional means.

Finally, sustainability must be considered before beginning any community-engaged research initiative. Community and philanthropic relationships facilitate the sustainability of WAI-Milwaukee's programming. Philanthropic funding draws other types of funding agencies to contribute to WAI-Milwaukee's infrastructure. Through leveraging philanthropic support in garnering other funding, infrastructure is sustained for retaining ongoing relationships and forging new relationships with community entities.

## Conclusion

WAI-Milwaukee applied ABCD principles to improve recruitment of African Americans into longitudinal research for ADRD. Initiatives grounded in ABCD approaches can potentially improve quality of care, reduce costs, decrease burden, improve quality-of-life and subsequently increase interest in research participation among African Americans. ABCD approaches can also potentially increase interest in therapeutic interventions among African-Americans that reduce the burden of ADRD, as exemplified by the family care coordination and development of the Amazing Grace Chorus Family Support Program. Finally, future models grounded in ABCD approaches can facilitate African-American recruitment into ADRD studies for the improvement of the field and decreasing disparities.

## Author Contributions

GG-H, SC, and RK drafted this manuscript and conducted the literature research. GG-H, SH, NN, and DE conceived the vision and implemented the asset-based community development approach for WRAP African-American recruitment for which this article is based. SH, NN, MS, SJ, and DE reviewed this article and provided content revisions. All authors provided their final agreement for submission of this manuscript.

### Conflict of Interest Statement

The authors declare that the research was conducted in the absence of any commercial or financial relationships that could be construed as a potential conflict of interest.
